# A self-made wire snare used in combination with a long transparent cap to remove a giant phytobezoar

**DOI:** 10.1055/a-2471-7861

**Published:** 2024-12-03

**Authors:** Lizhi Yi, Huarong Qiu, Ke Liu, Yanhong Ge, Zhengyu Cheng

**Affiliations:** 1 66561 Gastroenterology, Peopleʼs Hospital of Leshan, Leshan, China

A 76-year-old man with a 3-year history of consumption of persimmons was admitted to our hospital because of a 6-month history of abdominal distension. We performed esophagogastroduodenoscopy and revealed a giant phytobezoar (about 4 × 6 cm) in his stomach ( Fig. 1 **a** ). Because of its large size, the phytobezoar could not be trapped using the maximal diameter snare that was available (3 cm in diameter). A self-made wire snare was therefore created using a zebra guidewire, and a long transparent cap, with an edge that protrudes approximately 12 mm beyond the tip of the gastroscope, was installed onto the gastroscope ( Video 1 ). At the second attempt, the phytobezoar was successfully trapped with the self-made wire snare and was then slowly pulled to the edge of the long transparent cap ( Fig. 1 **b** ). Next, we continued pulling on the snare to cold-cut the phytobezoar with the assistance of the cap ( Fig. 1 **c** ). The procedure was repeated until the phytobezoar had been cut into several small pieces, all of which were then successfully removed ( Fig. 2 ). The patient did not feel any discomfort postoperatively and was discharged the following day.

**Fig. 1:**
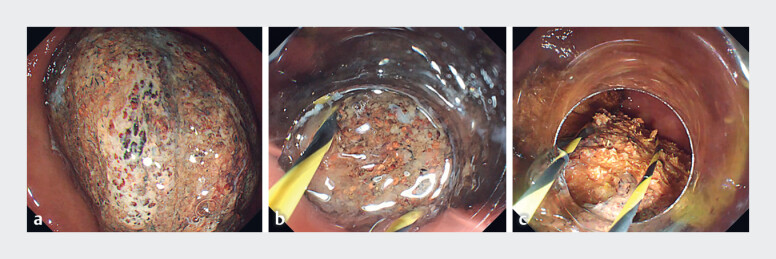
Endoscopic views showing: **a** a giant phytobezoar in the stomach; **b** the phytobezoar being trapped by the self-made wire snare and pulled into the long transparent cap; **c** the phytobezoar being cut into pieces.

A self-made wire snare is used in combination with a long transparent cap to successfully remove a giant phytobezoar.Video 1

**Fig. 2:**
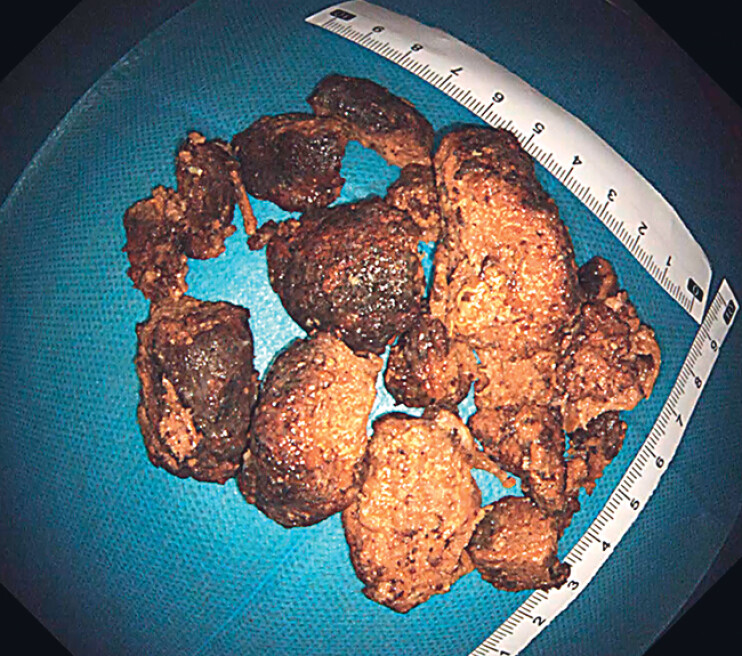
Photograph of the phytobezoar pieces after their successful removal.

Endoscopic fragmentation is a common strategy for the treatment of gastric phytobezoars 1 . A cap-assisted cold snare technique has been reported to be effective for the removal of giant phytobezoars 2 ; however, a snare that has a diameter larger than the phytobezoar may not be commonly available in clinical practice. The long transparent cap that we used was better at keeping the tip of the endoscope from being touched and damaged by the phytobezoar than the traditional transparent cap would have been. We show that a self-made wire snare combined with a long transparent cap can be used to successfully remove a giant phytobezoar using the cap-assisted technique.

Endoscopy_UCTN_Code_TTT_1AO_2AL

## Notes

Endoscopy E-Videos https://eref.thieme.de/e-videos
*E-Videos* is an open access online section of the journal *Endoscopy* , reporting on interesting cases and new techniques in gastroenterological endoscopy. All papers include a high-quality video and are published with a Creative Commons CC-BY license. Endoscopy E-Videos qualify for HINARI discounts and waivers and eligibility is automatically checked during the submission process. We grant 100% waivers to articles whose corresponding authors are based in Group A countries and 50% waivers to those who are based in Group B countries as classified by Research4Life (see: https://www.research4life.org/access/eligibility/ ). This section has its own submission website at https://mc.manuscriptcentral.com/e-videos .
